# Weighted Single-Step Genome-Wide Association Study of Semen Traits in Holstein Bulls of China

**DOI:** 10.3389/fgene.2019.01053

**Published:** 2019-10-25

**Authors:** Hongwei Yin, Chenghao Zhou, Shaolei Shi, Lingzhao Fang, Jianfeng Liu, Dongxiao Sun, Li Jiang, Shengli Zhang

**Affiliations:** ^1^National Engineering Laboratory for Animal Breeding, Key Laboratory of Animal Genetics, Breeding, and Reproduction, Ministry of Agriculture, College of Animal Science and Technology, China Agricultural University, Beijing, China; ^2^Department of Animal and Avian Sciences, University of Maryland, College Park, College Park, MD, United States

**Keywords:** semen traits, weighted single-step genome-wide association studies, Chinese Holstein, window regions, candidate genes

## Abstract

Efficient production of high-quality semen is a crucial trait in the dairy cattle breeding due to the widespread use of artificial insemination. However, the genetic architecture (e.g., distributions of causal variants and their corresponding effects) underlying such semen quality traits remains unclear. In this study, we performed genome-wide association studies to identify genes associated with five semen quality traits in Chinese Holstein population, including ejaculate volume, progressive sperm motility, sperm concentration, number of sperm, and number of progressive motile sperm. Our dataset consisted of 2,218 Holstein bulls in China with full pedigree information, representing 12 artificial insemination centers, with 1,508 genotyped using the Illumina BovineSNP50 BeadChip. We used a weighted single-step genome-wide association method with 10 adjacent Single nucleotide polymorphisms (SNPs) as sliding windows, which can make use of individuals without genotypes. We considered the top 10 genomic regions in terms of their explained genomic variants as candidate window regions for each trait. In total, we detected 36 window regions related to one or multiple semen traits across 19 chromosomes. Promising candidate genes of *PSMB5*, *PRMT5*, *ACTB*, *PDE3A*, *NPC1*, *FSCN1*, *NR5A2, IQCG, LHX8*, and *DMRT1* were identified in these window regions for these five semen traits. Our findings provided a solid basis for further research into genetic mechanisms underlying semen quality traits, which may contribute to their accurate genomic prediction in Chinese Holstein population.

## Introduction

The fertility of dairy cattle has decreased over recent decades due to highly intensive selection for milk production (Royal et al., 2000; Lucy, 2001; Walsh et al., 2011). Male fertility, as represented by the ability of the sperm to fertilize and activate an egg to ensure the normal embryo development, is vitally important for effective and efficient production of cattle (Kaya and Memili, 2016). Male fertility can be measured directly from individuals or indirectly from females. Unlike many male fertility traits measured based on the records of females (e.g., sire conception rate and daughter pregnancy rate), semen traits are measured directly in males.

Semen traits such as progressive sperm motility (SM) and ejaculate volume (VE) are complex and affected by genetic factors (Hering et al., 2014a; Hering et al., 2014b; Gottschalk et al., 2016; Qin et al., 2017; Sato et al., 2018) as well as by non-genetic factors such as handler, season, interval between ejaculations, and age (Mathevon et al., 1998; Fuerst-Waltl et al., 2006). The maturation of sperm in mammals requires cooperation among many genes and cell types, including germ cells, Leydig cells, and Sertoli cells (Sarkar et al., 2016). Mutations in genes related to spermatogenesis and sperm maturation may lead to reduction of semen quality and fertility (Ballow et al., 2006; Zhang et al., 2016). It is difficult to select animals directly based on their semen phenotypes due to the low to moderate heritability of these traits (0.04–0.30) (Gredler et al., 2007; Druet et al., 2009). Therefore, many studies (Suchocki and Szyda; Hering et al., 2014a; Hering et al., 2014b; Qin et al., 2017) have focused on identifying genes and genetic markers associated with bovine semen traits, in order to understand their genetic architectures of these traits. In other species, such as boars and stallion, several genome-wide association studies (GWAS) in recent years have been performed to detect functional genes for the semen traits (Gottschalk et al., 2016; Marques et al., 2018). However, the population sizes analyzed in these studies were not large (139–900), with low statistical power to detect causal genes. Since few genes were shared among these studies, the genetic architecture underlying semen traits still remains elusive.

In our previous study, a single-SNP GWAS was performed for five semen traits in a population of 692 animals (Qin et al., 2017). Due to the small number of animals with both genotype and phenotype, de-regressive proofs (DRPs) were calculated and used as response variables in the GWAS model, and a genome-wide Bonferroni was applied to avoid false positives. The results indicated that only 19 SNPs were significantly associated with five semen traits.

In order to study the genetic basis of the semen traits, a weighted single-step GWAS (WssGWAS) was performed in a larger Chinese Holstein bull population. Recent studies have shown that WssGWAS can improve precision in estimating SNP effects and perform better than traditional methods under certain conditions, including with small sample sizes with both genotypes and phenotypes where traits are regulated by loci with large effects (Wang et al., 2014; Marques et al., 2018). It also allows for different weights of various SNPs (Aguilar et al., 2010; Wang et al., 2014; Marques et al., 2018). In addition, although estimated breeding values (EBVs) or de-regressed EBV (DRP) (Garrick et al., 2009) has often been used to represent phenotypes in GWAS, EBV cannot utilize all information (e.g., genotypes) and DRP may lead to losses of accuracy and biases due to inflation (Vitezica et al., 2011; Ricard et al., 2013). As such, we instead used the raw phenotypes to conduct GWAS while controlling for all known systemic effects. We identified some novel candidate genes and window regions related to five semen traits of Holstein bulls in China. Our results provide insights into the genetic basis of semen traits in dairy cattle.

## Methods

### Ethics Statement

All procedures pertaining to the handling of experimental animals were conducted in accordance with and approved by the Animal Welfare Committee of China Agricultural University (Permit Number: DK996). All efforts were made to minimize discomfort and suffering.

### Phenotype, Genotype, and Pedigree

**Figure 1:**
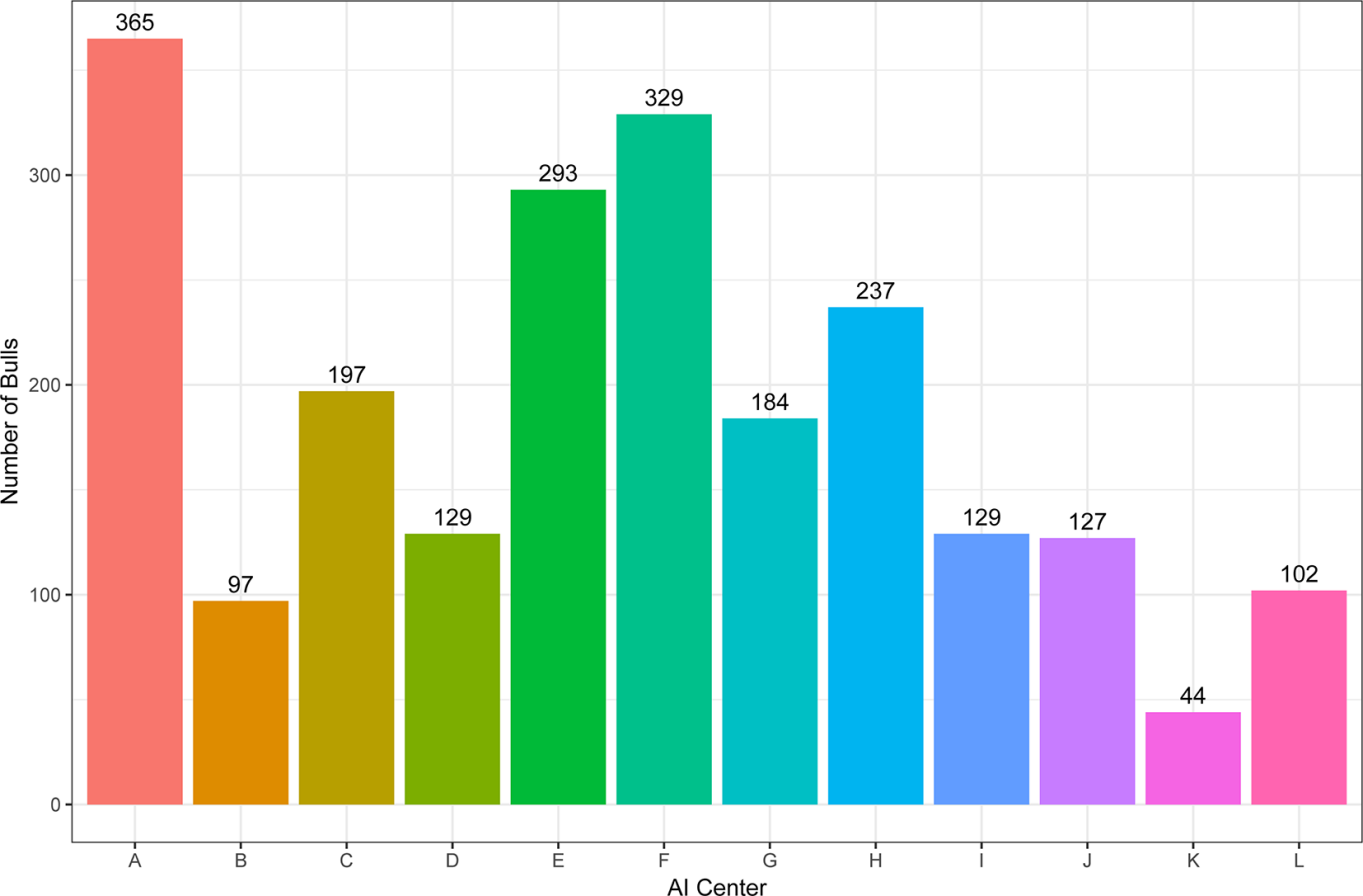
The distribution of bulls across artificial insemination (AI) centers.

**Figure 2:**
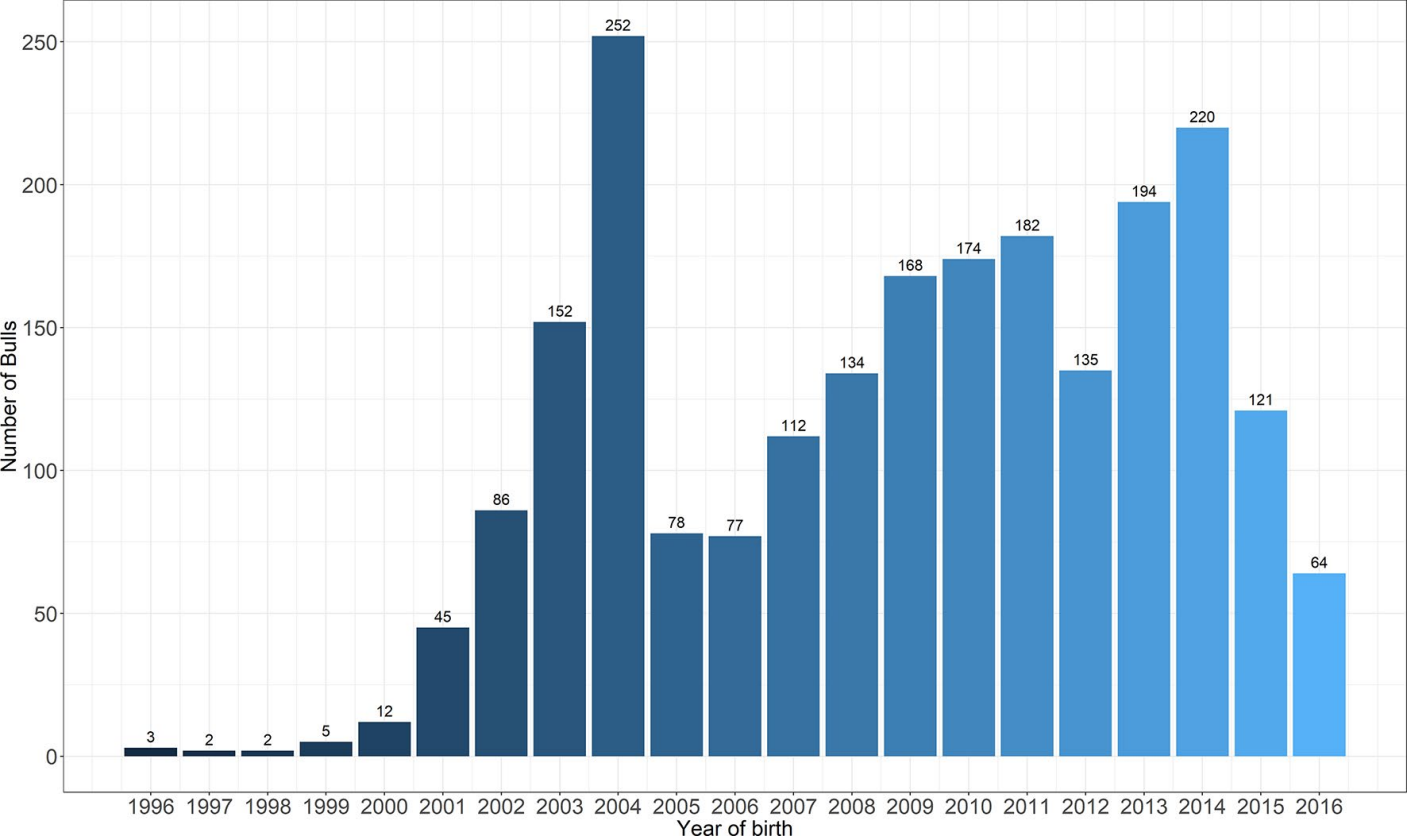
The distribution of bulls across the years of birth.

A total of 2218 Holstein bulls born between 1996 and 2016 were represented in semen samples and phenotypes, from 12 artificial insemination (AI) centers across China where routine semen testing has been carried out since 1995. Distributions of bulls’ birth year and location are given in **Figures 1** and **2**, respectively. At each AI center, artificial ejaculations were conducted two times per day by semen handlers, and the interval between the two continuous semen collections for each bull was 30 min to 1 h. The management of AI centers and semen collection routines were consistent at each AI centers, which was ruled by the Department of Agriculture in China. These AI centers had frequent gene communications, and some bulls were born and grow up in one AI center, but were collected semen in other AI center. Five semen traits were recorded for each individual. VE in ml was read directly from a graduated collection tube. Progressive SM was estimated as the percentage of forward-moving sperm, evaluated using a microscope by an experienced technician. Sperm concentration (SC) was measured using a spectrophotometer as 10^9^ spermatozoa per ml. Number of sperms (NSP) per ejaculate was calculated by multiplying VE and SC, and the number of motile sperm (NMSP) was obtained by multiplying NSP and SM. After removal of the error data [VE ranging from 0 to 30, SC ranging from 0 to 40, motility of sperm (MS) ranging from 0 to 100%], we yielded phenotypic records of 527,207 records of VE, 527,231 records of SC, 521,150 records of MS and NMSP, and 522,906 records of NSP. The means, standard deviations, and minimum and maximum values of these traits are shown in **Table 1**.

**Table 1:** Descriptive statistics of five semen traits.

Traits	Number of records	Number of bulls	Mean	Median	Min	Max	SD
**VE**	527,207	2,218	6.87	6.50	0.1	30.00	3.07
**SC**	521,510	2,218	12.07	11.80	0.01	37.80	4.66
**MS**	527,231	2,218	0.66	0.70	0.00	0.98	0.16
**NMSP**	522,906	2,218	57.57	49.04	0.00	469.25	39.99
**NSP**	521,150	2,218	83.71	75.40	0.02	572.25	48.94

Recorded for each ejaculate [VE, ejaculate volume (ml); SM, progressive sperm motility (%); SC, sperm concentration (109); NSP, number of sperm per ejaculate (109); NMSP, number of motile sperm (109)].

DNA was extracted from 1508 semen samples using a standard phenol–chloroform method. Illumina BovineSNP50 BeadChip versions 1 and 2 (Illumina Inc., USA) were used to genotype these individuals. For filling the missing genotypes, genotype imputation was carried out using BEAGLE software version 3.3.1 (Browning and Browning, 2007), and 52,886 SNPs on 29 autosomes were obtained after imputation. Quality control was performed where SNPs were excluded from further analysis when genotype call rate was <90%, minor allele frequency was <0.01, or Hardy–Weinberg equilibrium statistics were <1-e6. A total of 44,074 SNPs in the bovine genome met the requirements and were used to conduct an association study. The bovine genome assembly UMD3.1 was used to identify SNP locations.

The complete pedigrees of all individuals included in this study comprised a total of 4,237 animals, which had 497 sires, 1,512 dams, and 2,695 bulls. The number of bulls with phenotypes was 2,218, and the number of bulls with both phenotypes and genotypes was 1,508.

### Statistical Analyses

Two main methods can be implemented to GWAS in semen traits. One fits individual SNP as a fixed effect in a mixed model that outputs *P* value to evaluate the importance of SNPs, and the other is a Bayesian method that utilizes all SNPs simultaneously as random effect and output SNPs effect to evaluate their importance (Wang et al., 2014). Recently, the single-step GWAS method was developed to integrate all information including genotypes, phenotypes, and pedigree information in one step using a matrix. Based on this, genomic estimated breeding value (GEBVs) of all animals were estimated by single-step genomic best linear unbiased prediction (ssGBLUP) and transformed into SNP effects. Finally, the percentage of genetic variance explained by SNP windows was calculated using sets of consecutive SNP effects. So we conducted a WssGWAS using BLUPF90 software family (Misztal et al., 2002). First, ssGBLUP (Legarra et al., 2014) was conducted using BLUPF90 (Misztal and Genetics, 2008), and then used to predict GEBV. SNP effects were calculated using postGSf90 in BLUPF90 software.

For each of the five semen traits, the animal model for sssGBLUP was as follows: y = **X**β + **Za** + **W**p + e, where y is the vector of the semen phenotype; β is the vector of fixed effects (combined effects of year and season collection, AI centers, interval between two subsequent semen collections, the number of sample collections on the respective collection day, and the covariates of age of the bulls at the time of collection in months); ais the vector of additive genetic effects with $a\sim N(0,H\sigma_{a}^{2})$, where $\sigma_{a}^{2}$ is the additive genetic variance; p is the vector of permanent environmental effects with $p\sim N(0,I\sigma_{p}^{2})$, where $\sigma_{p}^{2}$ is the permanent environmental variance; e is the vector of random residuals with $e\sim N(0,I\sigma_{e}^{2})$, where $\sigma_{e}^{2}$ is the residual variance; and **X**, **Z**, and **W** are the design matrices of β, a, and p, respectively. **I** is an identity matrix, and **H** ^–1^ is created as follows: $H^{-1}=A^{-1}+\left[\begin{array}{cc} 0 & 0\\ 0 & G^{-1}-A_{22}^{-1} \end{array}\right]$, where **A**_22_ is the numerator relationship matrix for genotyped animals (Aguilar et al., 2010) and **A** is the numerator relationship matrix of all pedigreed animals and **G** is the genomic relationship matrix (Vanraden, 2008). The matrix of **G** was calculated as follows: **G** = **ZDZ**′*q*, where **Z** is the marker matrix coded for allele frequencies (*aa* = 0, *Aa* = 1and *AA* = 2), **D** is a diagonal matrix of weights for SNP variances (initially **D** = **I**), and *q* is a weighting factor. The weighting factor can be derived based on SNP frequencies (Vanraden, 2008).

SNP effects and weights for WssGWAS were calculated as the following steps (Wang et al., 2012):

1. Let **D** = **I** in the first iteration.
2. Calculate **G** = **ZDZ**′*q*.
3. Calculate the GEBVs using ssGBLUP for entire dataset.
4. GEBV were converted to estimates of SNP effects $\left(\hat{u}\right)$: $\hat{u}=\lambda DZ^{'}G_{}^{-1}{\hat{a}}_{g}$, where ${\hat{a}}_{g}$ is the GEBV of animals genotyped.
5. The weight for individual SNP was calculated as $d_{i}={\hat{u}}_{i}^{2}2p_{i}\left(1-p_{i}\right)$, where *i* is the *i*th SNP.
6. Normalize SNP weights to keep total genetic variance constant.
7. Return to step 2.

We calculated the SNP weights iteratively looping through steps 2–6. Iterations increase the weights of SNPs with large effects and absorb those with small effects. The procedure was run for different iterations based on accuracy (Wang et al., 2014), and one iteration was selected for our study. The percentage of genetic variance explained by the *i*th SNP window was calculated as follows: $\frac{Var\left(a_{\text{i}}\right)}{\sigma_{a}^{2}}\times 100\%=\frac{Var\left(\sum_{j=1}^{x}Z_{j}{\hat{u}}_{j}\right)}{\sigma_{a}^{2}}\times 100\%$, where *a* *_i_* is the genetic value of the *i*th SNP window that consists of a region of consecutive SNPs; $\sigma_{a}^{2}$ is the total additive genetic variance; **Z** *_j_* is the vector of gene content of the *j*th SNP for all individuals; and ${\hat{u}}_{j}$ is the effect of the *j*th SNP within *i*th window.

Manhattan plots showing these windows were created using CMplot package of the R software.

### *In Silico* Functional Analyses

We used the BioMart web to map these top 10 regions to bovine genome in version of UMD 3.1 (Smedley et al., 2009) and got the gene names of these genes. Gene ontology (GO) enrichment analysis of genes in the top 10 regions of the five semen traits was performed in DAVID database of version 6.8 (Huang Da et al., 2009) with default parameter. GO terms [Biological Process (BP), Cellular Component (CC), and Molecular Function (MF)] with *P* values < 0.01 were considered to be significantly enriched.

We also used the web of genecards (https://www.genecards.org/) to investigate the gene function based on orthologous genes of human.

## Results

**Figure 3:**
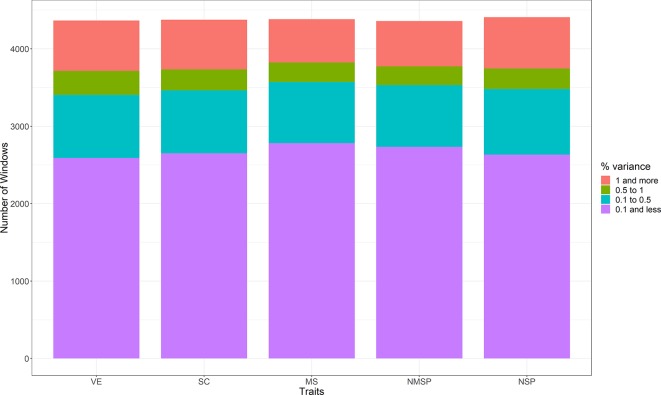
The distribution of the four classes in five semen traits (the regions explained genetic variance: >1%, 0.5%–1%, 0.1%–0.5%, and <0.1%. VE, ejaculate volume; SM, progressive sperm motility; SC, sperm concentration; NSP, number of sperm per ejaculate; NMSP, number of motile sperm).

The proportions of genetic variance explained by each window for the five traits were obtained by WssGWAS. We divided the regions into four classes according to their explained genetic variance, as >1%, 0.5%–1%, 0.1%–0.5%, and <0.1% (**Figure 3**). Most of the window regions explained less than 0.1% of the genetic variance, and the number of window regions that explained more than 1% of the genetic variance accounted for 15.0% of VE, 14.7% of SC, 12.8% of MS, 13.4% of NMSP, and 15.0% of NSP. For each trait, the top 10 ranked windows by their explained genetic variance are in **Table 2**, and the details of these window regions for the five traits are further described as follows.

**Table 2:** Individual and overlapping of the top 10 window regions (UMD3.1) among five semen traits.

Chr	Window region (Mb)	Var (%)VE	Var (%)SC	Var (%)MS	Var (%)NMPS	Var (%)NSPE	Genes
**1**	108.74-109.10	–	–	2.93	–	–	*IQCJ*
**1**	109.18–109.52	–	–	2.09	–	–	–
**2**	56.5–56.9	3.34	–	–	–	–	*ENSBTAG00000024632*
**2**	65.04–65.35	–	3.61	–	–	–	*NCKAP5,LYPD1,ENSBTAG00000047466*
**2**	4.2–4.57	–	2.94	–	–	–	*UGGT1,SAP130,AMMECR1L,POLR2D*
**3**	71.6–72.04	–	4.59	3.25	2.12	1.80	–
**3**	76.57–76.85	–	2.21	–	–	–	–
**3**	69.91–70.27	–	–	–	2.16	–	*LHX8,TYW3,CRYZ*
**4**	64.55–65.17	–	–	3.77	–	–	*PDE1C,PPP1R17*
**5**	113.91–114.47	6.92	–	–	14.88	8.67	*SERHL2,RRP7A,POLDIP3,CYB5R3,ARFGAP3,PACSIN2,A4GALT,ENSBTAG00000030199,ENSBTAG0000004557,ENSBTAG00000037110*
**5**	113.47–113.87	3.49	–	–	4.77	4.51	*SEPT3,WBP2NL,NAGA,PHETA2,SMDT1,NDUFA6,MGC127055,CYP2D14,TCF20,NFAM1,bta-mir-2442,ENSBTAG00000038146,ENSBTAG00000037632,ENSBTAG00000046721*
**5**	89.79–90.27	–	–	16.82	–	–	*ENSBTAG00000044467*
**5**	89.29–89.68	–	–	2.29	–	–	*SLCO1A2,SLCO1B3,ENSBTAG00000016876,ENSBTAG00000042573,SLCO1C1,PDE3A,ENSBTAG00000029126*
**6**	0.62–0.94	10.01	–	–	–	–	–
**8**	43.78–44.28	–	–	–	2.52	1.91	*DMRT2,ENSBTAG00000043651,DMRT3, DMRT1, KANK1*
**9**	95.19–95.67	–	–	–	2.70	2.55	*ARID1B,TMEM242, ZDHHC14,bta-mir-2481*
**10**	21.52–21.87	1.55	–	–	–	–	*SLC7A8,ENSBTAG00000043704,ENSBTAG00000042621,CEBPE,C10H14orf119,ACIN1,CDH24,PSMB11,PSMB5,C10H14orf93,AJUBA,HAUS4,PRMT5,RBM23,REM2,LRP10,MMP14,MRPL52,SLC7A7,OXA1L*
**10**	50.75–51.1	–	–	–	1.49	–	*FAM81A,MYO1E*
**11**	18.22–18.48	–	19.55	–	–	–	*ENSBTAG00000045038*
**11**	45.35–45.85	–	–	1.76	–	–	*ST6GAL2,UXS1,ENSBTAG00000044532, C11H2orf40, NCK2*
**15**	85.05–85.26	3.75	–	–	–	–	*ENSBTAG00000048316*
**16**	37.12–37.36	2.33	–	–	–	–	*DPT*
**16**	80.56–80.9	2.30	–	–	–	–	*NR5A2*
**16**	75.75–76.06	–	2.03	–	–	–	*bta-mir-205,ENSBTAG00000044497*
**17**	66.83–67.22	–	1.19	–	–	–	*WSCD2,ENSBTAG00000047446*
**17**	42.47–42.81	–	–	16.98	2.42	–	*GRIA2,ENSBTAG00000047318*
**19**	60.01–60.36	–	–	1.87	–	–	–
**19**	30.22–30.57	–	–	–	–	1.53	*MYH3,SCO1,ADPRM,TMEM220,PIRT*
**20**	4.96–5.3	–	–	–	2.23	1.46	*BOD1*
**20**	34.05–34.39	–	–	–	–	1.51	–
**21**	25.48–25.85	–	–	1.95	–	–	*BTBD1,MORF4L1, CTSH,RASGRF1*
**22**	14.06–14.39	1.63	–	–	–	–	–
**22**	60.82–61.07	–	1.85	–	–	–	*PLXNA1,CHCHD6*
**24**	33.25–33.69	1.68	–	–	5.77	4.48	*LAMA3,ENSBTAG00000039850,RF00026,ANKRD29,NPC1,RMC1,RIOK3,TMEM241,CABLES1*
**25**	4.82–5.13	–	3.49	–	–	–	–
**25**	39.26–39.54	–	1.43	–	–	–	*RNF216,FSCN1,ENSBTAG00000047781,ACTB,FBXL18,TNRC18,SLC29A4*

Chr, chromosome; window region: position of window region. Recorded for each ejaculate (VE, ejaculate volume; SM: progressive sperm motility; SC, sperm concentration; NSP, number of sperm per ejaculate; NMSP, number of motile sperm). Var: the percentage of genetic variance explained by the window region; Genes: the genes located within the window region. –: no genes located in the window region or not the top 10 ranking windows for the semen trait. Var(%): the percentage of the explained variance.

### VE

**Figure 4:**
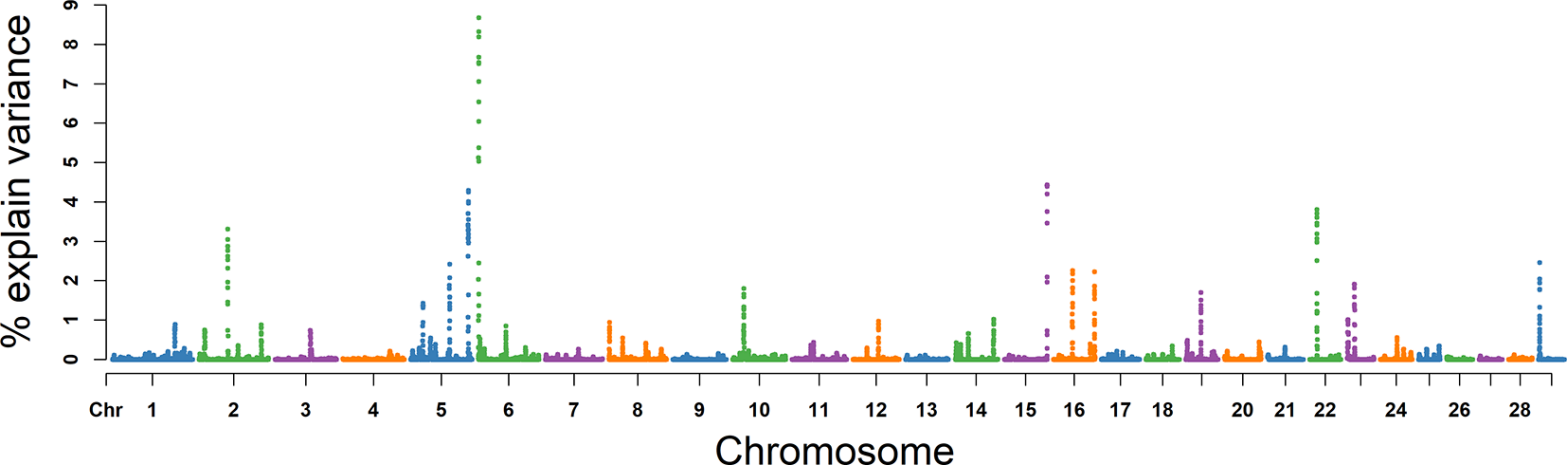
GWAS results of ejaculate volume (VE) in Holstein bulls of China. Each dot represents one SNP. The X-axis represents 29 autosomes, respectively. The Y-axis represents the percentage of genetic variance explained by SNP.

The explained variance and *P* values for each SNP in VE are shown in **Figure 4** and **Figure S1**. The most important window for VE that explained 10.00% of the genetic variance was on BTA 6. The top 10 ranked windows are summarized in **Table 3**. These windows were located on BTA 2, 5, 6, 10, 15, 16, 22, and 24, in descending order of rank. The genetic variance explained by the top 10 windows ranged from 1.55% to 10.00%. Fifty-seven genes were involved in these selected regions (**Table 2**).

**Table 3:** Candidate genes for five semen traits.

Traits	Gene	Chr	Start	End	Windows
**VE**	PSMB5	10	21658161	21663680	TOP5
**VE**	NR5A2	16	80751686	80877243	TOP7
**VE**	PRMT5	10	21752766	21760473	TOP5
**VE**	NPC1	24	33438419	33485514	TOP4
**SC**	FSCN1	25	39292721	39302192	TOP6
**SC**	ACTB	25	39343633	39347044	TOP6
**MS**	PDE3A	5	89554512	89620721	TOP6
**MS**	IQCJ	1	109080711	109102233	TOP5
**NMSP**	NPC1	24	33438419	33485514	TOP1
**NMPS**	LXH8	3	69890357	69916293	TOP10
**NMSP**	DMRT1	8	43916605	43972570	TOP3
**NSP**	NPC1	24	33438419	33485514	TOP1
**NSP**	LXH8	3	69890357	69916293	TOP10
**NSP**	DMRT1	8	43916605	43972570	TOP3

Recorded for each ejaculate (VE, ejaculate volume; MS, progressive sperm motility; SC, sperm concentration; NSP, number of sperms per ejaculate; NMSP, number of motile sperm). Chr, chromosome; Start, the start SNP number of the gene; End, the end number SNP of the gene.

### SC

**Figure 5:**
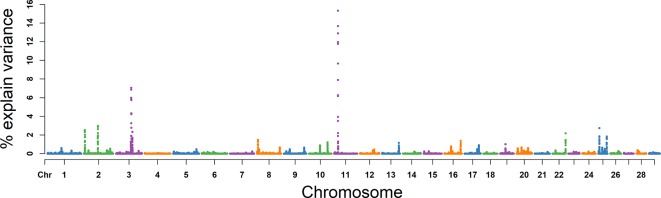
GWAS results of sperm concentration (SC) in Holstein bulls of China. Each dot represents one SNP. The X-axis represents 29 autosomes, respectively. The Y-axis represents the percentage of the explained variance by SNP.

For SC, the explained variance and *P* values for each SNP are shown in **Figure 5** and **Figure S2**. The top 10 ranked windows explaining the genetic variance ranged from 1.19% to 19.55% (**Table 2**). These windows were located on BTA 2, 3, 11, 16, 17, 22, and 25, in descending order. The most important window for SC, explaining 19.55% of genetic variance, is located on BTA 11 (**Figure 5**). There were 21 genes in these selected regions (**Table 2**).

### MS

**Figure 6:**
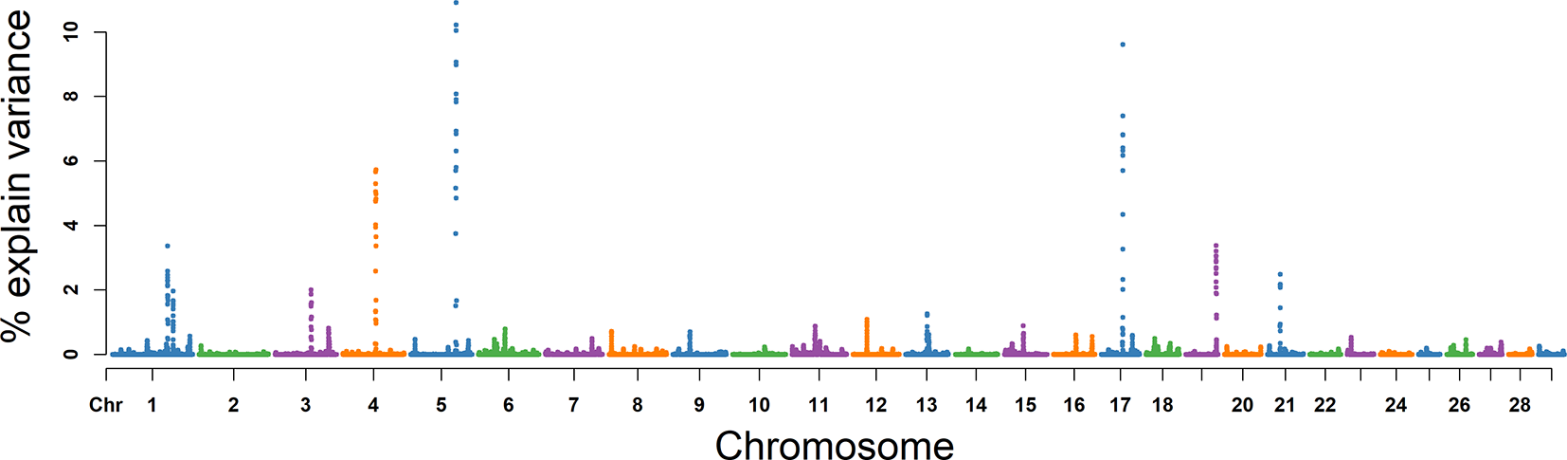
GWAS results of progressive sperm motility (MS) in Holstein bulls of China. Each dot represents one SNP. The X-axis represents 29 autosomes, respectively. The Y-axis represents the percentage of the explained variance by SNP.

For MS, the explained variance and *P* values for each SNP in VE are shown in **Figure 6** and **Figure S3**. The two most important windows were located on BTA 17 and BTA 5, which respectively explained 16.98% and 16.82% of the genetic variance (**Table 2**). The genetic variance explained by the top 10 windows ranged from 1.76% to 16.98% (**Table 2**). These windows were located on BTA 1, 3, 4, 5, 11, 17, 19, and 21, in descending order. A total of 22 genes were clustered within these regions (**Table 2**).

### NMSP

**Figure 7:**
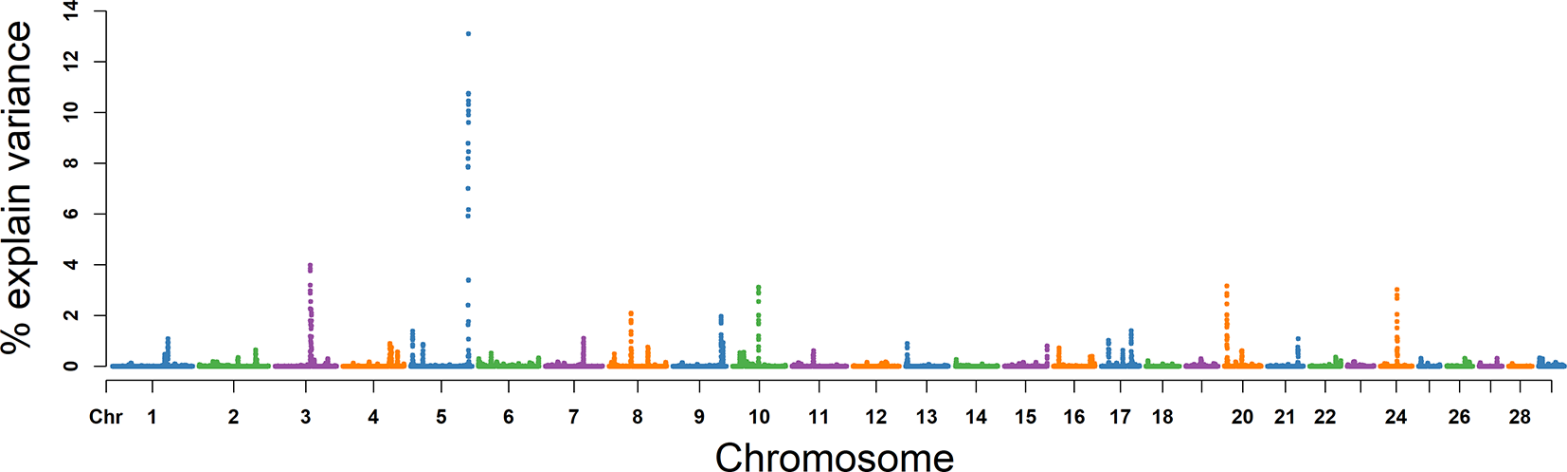
GWAS results of number of motile sperm (NMSP) in Holstein bulls of China. Each dot represents one SNP. The X-axis represents 29 autosomes, respectively. The Y-axis represents the percentage of the explained variance by SNP.

The explained variance and *P* values for each SNP in NMSP are shown in **Figure 7** and **Figure S4**. The most important windows for NMSP were on BTA 5 and explained 14.88% of the genetic variance (**Table 2**). The genetic variance explained by the top 10 ranked windows ranged from 1.59% to 14.88% (**Table 2**), which were located on BTA 3, 5, 8, 9, 10, 17, 20, and 24, in descending order. A total of 50 genes were involved in the selected region (**Table 2**).

### NSP

**Figure 8:**
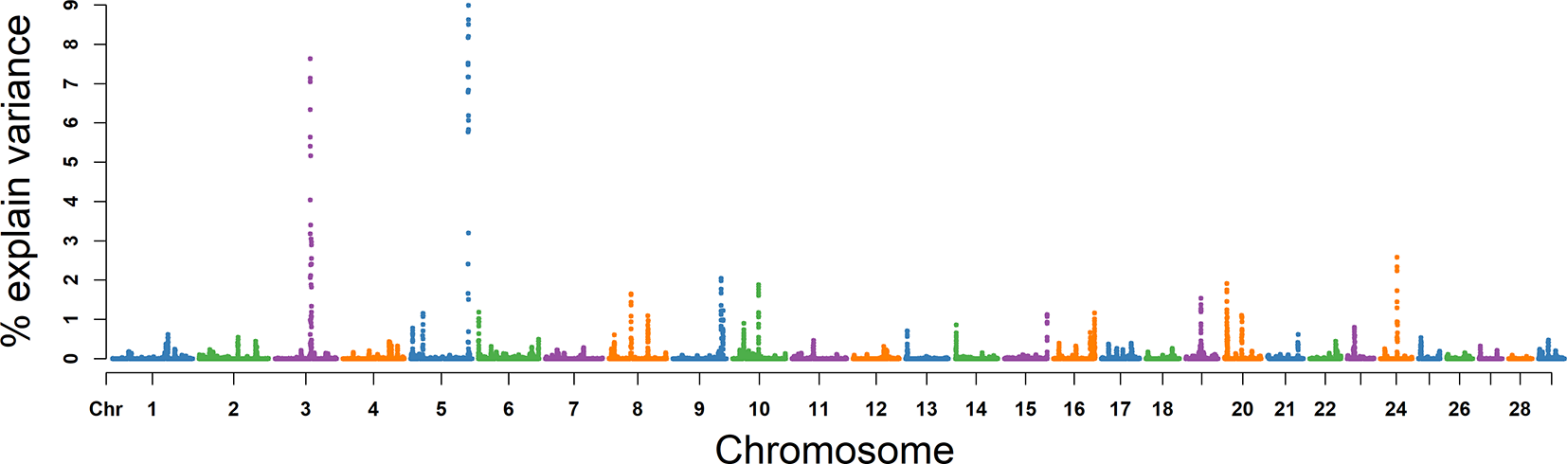
GWAS results of number of sperms per ejaculate (NSP) in Holstein bulls of China. Each dot represents one SNP. The X-axis represents 29 autosomes, respectively. The Y-axis represents the percentage of the explained variance by SNP.

The explained variance and *P* values for each SNP in NSP are shown in **Figure 8** and **Figure S5**. The most important window for NSP on BTA 5 explained 8.67% of the genetic variance (**Table 2**). The genetic variance explained by the top 10 ranked windows ranged from 1.46% to 8.67% (**Table 2**), located on BTA 3, 5, 8, 9, 10, 19, 20, and 24, in descending order. There were 51 genes in these selected regions (**Table 2**).

In total, we identified 36 window regions across 19 chromosomes, out of which 8 window regions were overlapped by at least two semen traits. On BTA 5, a window region of 113.47–113.87 Mb was shared by VE, NMSP, and NSP, and 12 genes were located in this region. Out of these 12 genes, *WBP2NL* was specifically highly expressed in the testes, according to the UniProt/SwissProt database (https://www.uniprot.org/uniprot/Q6ICG8#expression). On BTA 8, a window region of 43.78–44.28 Mb was shared by NMSP and NSP, with five genes located there. Of these, *DMRT1* was a cell-identifying gene in the testis cord and seminiferous tubules, according to LifeMap Discovery (https://discovery.lifemapsc.com/in-vivo-development/testis). On BTA 24, the window region 33.25–33.69 Mb was shared by VE, NMSP, and NSP, and nine genes were located there. Of these, the expression of *NPC1* was related to the testis interstitium according to LifeMap Discovery (https://discovery.lifemapsc.com/in-vivo-development/testis). 

### GO Analyses and Functional Annotation

The GO enrichment analysis of the 10 top regions for the five semen traits revealed 11 significant GO terms, including biological process, cellular component, and molecular function. The details of the significant GO terms are shown in **Additional File 1: Table S1**. Of interest was the GO term for steroid metabolic process (GO: 0008202, *P* = 0.001), which is likely to be related to semen traits. Six genes were in this GO term, including *CYB5R3, SLCO1B3, NPC1, SLCO1A2, SLCO1C1,* and *NR5A2*. It is worth noting that the *DMRT1* gene was in the GO term of male germ cell proliferation (GO: 000217) and spermatogenesis (GO: 0007283), indicating that *DMRT1* is a promising candidate gene.

Based on analysis above, we concluded 10 promising candidate genes for these five semen traits (**Table 3**).

## Discussion

In WssGWAS, different weights were given to SNPs according to their importance, and linkage disequilibrium made the consecutive SNP window method more effective at detecting Quantitative Trait Loci (QTL) compared with a single-SNP method (Marques et al., 2018). As a result, we can detect important QTLs for each trait. Because this method can utilize both genotyped and un-genotyped individuals in the pedigree, 710 ungenotyped animals were used in this study, and 4237 animals in the pedigree file were used to calculate GEBV and estimate SNP effects. These ungenotyped individuals can supply additional information to improve the statistical power of QTL detection. Sample size can influence the power of GWAS (Marques et al., 2018).

In our previous study, a single-SNP GWAS was performed for five semen traits in a population of 692 animals (Qin et al., 2017). The results indicated that only 19 SNPs were significantly associated with five semen traits. In these regions reported by our previous study, 22 novel candidate genes were identified on chromosomes 1, 5, 6, 7, 15, 17, 23, and 27 (Qin et al., 2017). In the present study, many more window regions affecting these traits were detected, and the explained genetic variance was overall up to 1%, which indicated that the WssGWAS was more effective in detecting window compared with single-SNP GWAS. Of the 36 windows, one was consistent with our previous GWAS results and included the two candidate genes *PDE3A* and *SLCO1C1* (Qin et al., 2017). *PDE3A* on BTA5 is a member of the phosphodiesterase family and is expressed in the post-acrosomal segment of the sperm head (Qin et al., 2017). It has also been reported that *PDE3A* plays an important role in regulating mammalian acrosome reaction, SM, and capacitation by signal transduction systems (Lefievre et al., 2002; Oatley et al., 2009). Furthermore, IQ motif containing G (*IQCG*) on BAT 1 was detected in our study, which was also identified in GWAS by Marques et al. (2018) for MS traits in boars. *IQCG* knockout mice can show total immobility and severe malformation of spermatozoa, due to disorganization of the sperm flagellum axoneme (Li et al., 2014). Moreover, mutations of the *IQCG* gene in mice can lead to spermiogenesis defects and incomplete sperm tail formation (Harris et al., 2013).

It presented that some detected top windows are associated with phenotypic variation in multiple traits. There are eight windows explaining more than 1% of the genetic variance overlapping for these semen traits. In particular, there are seven overlapping window regions between NMSP and NSP. It has been suggested that high genetic correlations (0.96) between NMSP and NSP were confirmed in our previous study (Yin et al., 2019), and our findings of overlapping window regions supported our findings of overlapping window regions. Of these, an important window region found on BTA 3 contained a promising candidate gene, *LIM homeobox 8* (*LHX8*). *LHX8*, preferentially expressed in testicular germ cells (Su et al., 2002), may play an important function in spermatogenesis. It may also play a role in the regulation of spermatogonial differentiation into spermatocytes and from spermatogenesis (Ballow et al., 2006; Pangas et al., 2006; Rossi et al., 2008).

Based on the results of GO analyses, we propose three promising candidate genes for VE, NMSP, and NSP. *NPC1* and *NR5A2* were included in the GO term of steroid metabolic process (GO: 0008202) (*P* = 0.001), which is the most important to semen traits due to their spermatogenesis function being regulated by steroid hormones, the most important of which is testosterone. *NPC intracellular cholesterol transporter 1 (NPC1)* on BTA 24 was located in an overlapping window region among VE, NMSP, and NSP. Lack of a functional *NPC1* protein can cause multiple defects in mouse sperm and result in sterility (Fan et al., 2006). The cholesterol trafficking protein Niemann–Pick C1 (NPC1) is required for *Drosophila* sp. spermatogenesis (Wang et al., 2011). The mutation of the *NPC1* gene in mice causes dysregulation of testicular cholesterol metabolic processes (Akpovi et al., 2014). *NPC1* plays a role in normal cholesterol homeostasis and is essential for normal adrenal development and function (Gevry and Murphy, 2002) and may play a role in spermatogenesis. *NR5A2* on BTA 16 was a candidate gene for VE, which induces the process of steroidogenesis and inhibits G9a-mediated histone methylation during spermatogenesis in mice (Liu et al., 2016) and may regulate spermatogenesis *via* steroidogenesis. Liver receptor homologue-1 (LRH), expressed from *NR5A2*, is produced in human steroidogenic tissues and activates transcription of genes encoding steroidogenic enzymes (Sirianni et al., 2002) and may play an important role in spermatogenesis. In addition, the expression of *NR5A2* is directly controlled by *DMRT1* (Krentz et al., 2013). The genes *DMRT1* on BTA 8 was in the GO term of male germ cell proliferation (GO: 000217) and spermatogenesis (GO: 0007283). *DMRT1* is located in an overlapping window region between NMSP and NSP, and is required for spermatogonial stem cell replenishment and maintenance in mice (Zhang et al., 2016). In mammals, the mitosis–meiosis transition of spermatogenesis is regulated by the transcription factor *DMRT1* (Nakagawa et al., 2017). The binding sites for pioneer factor *DMRT1* are strikingly enriched in the open chromatin of human adult spermatogonial stem cells (Guo et al., 2017) and may regulate their differentiation.

By integrating analysis with the biological functions described in previous studies, we also identified four promising candidate genes, *PSMB5, PMRT5, FSCN1,* and *ACTB*. *PSMB5* and *PMRT5* on BTA 10 were promising candidate genes for VE. *PSMB5* was found to be associated with *RhoS* in a series of stage-specific spermatogenic cells as a new member of the Rho GTPase family in spermatogenic cells, which have been confirmed to be essential for mammalian spermatogenesis (Zhang et al., 2010). Loss of *PRMT5* in early PGCs leads to female and male sterility (Kim et al., 2014). A protein arginine methyltransferase is encoded by *PMRT5*, which has been demonstrated during embryonic stages to play important roles in germ cell development (Wang et al., 2015). *B-actin (ACTB)* on BTA 25 was identified as a promising candidate gene for MS. *ACTB* is expressed in sperm and is distributed in the acrosomal and postacrosomal regions of ejaculated spermatozoa, where it is potentially involved in membrane changes during the acrosome reaction with important implications to sperm function (Casale et al., 1988). Actin polymerization during capacitation and acrosome reaction is important for the fertilization process (Castellani-Ceresa et al., 1993). The exposure of actin on the surface of the human sperm head is significantly correlated with sperm morphology in zona binding, capacitation, and semen, and may provide a useful marker for sorting sperm cells with good potential to fertilize (Liu et al., 2005). The *B-actin (ACTB)* gene regulates the process from spermatogenesis to fertilization (Lin et al., 2006). On BTA 25, we identified *FSCN1* as a promising candidate gene for SC. FSCN1 protein is found at Sertoli cell junctions and in the neck region of elongate spermatid (Tubb et al., 2002). *FSCN1* is expressed throughout spermatogenesis and may be critical for normal sperm morphology and SM (Cheng et al., 2007). Taken together, these findings provide confirmatory evidence for previous studies, and further study and integration of these findings will surely promote a better understanding of the genetic architecture of semen traits in Holstein bulls.

## Conclusion

Our study revealed 36 window regions associated with five semen traits in the Chinese Holstein bull population using WssGWAS. GO term analysis suggested that *NR5A2, NPC1*, and *DMRT1* genes could be considered promising candidate genes for semen traits. Based on previous research into related semen traits and the biological functions of these genes, seven further promising candidate genes for semen traits are proposed, including *LHX8*, *PDE3A*, *IQCJ*, *PSMB5*, *PRMT5*, *ACTB*, and *FSCN1*. These findings lay a solid foundation for research into the genetic mechanism of semen traits and provide information for marker-assisted selection or genome selection for semen production traits in Holstein bulls.

## Data Availability Statement

The datasets generated for this study can be found in https://figshare.com/articles/semen_trait_gwas/7562510.

## Ethics Statement

All procedures pertaining to the handling of experimental animals were conducted in accordance with and approved by the Animal Welfare Committee of China Agricultural University (Permit Number: DK996). All efforts were made to minimize discomfort and suffering.

## Author Contributions

SZ and JL designed and supervised the study. HY and CZ contributed to genomic DNA extraction and conducted GWAS analysis, HY and CZ contributed to population construction and statistical analysis with help from JL and DS. SS contributed to genomic DNA extraction, phenotypes collection, and sample collection. HY drafted the manuscript, which was critically remarked by LF, JL, DS, and SZ. All authors read and approved the final manuscript.

## Funding

This work is supported by the 863 project (2013AA102504); the National Science and Technology Programs of China (2011BAD28B02); National Key Technologies R&D Program (2012BAD12B01); Beijing Dairy Industry Innovation Team, China Agricultural Research System (CARS-37); and Xinjiang Province Key Technology Integration and Demonstration Program (201230116). We are deeply grateful to all donors who participated in this program.

## Conflict of Interest

The authors declare that the research was conducted in the absence of any commercial or financial relationships that could be construed as a potential conflict of interest.

The reviewer GM declared a past co-authorship with one of the authors LF to the handling editor.

## Supplementary Material

Additional File 1: Table S1 The significant GO term of the 10 top regions for the five semen traits (*P < 0.01*).

Figure S1 GWAS results of ejaculate volume (VE) in Holstein bulls of China. Each dot represents one SNP. The X-axis represents 29 autosomes, respectively. The Y-axis represents the log10 of the P-Value by SNP.

Figure S2 GWAS results of sperm concentration (SC) in Holstein bulls of China. Each dot represents one SNP. The X-axis represents 29 autosomes, respectively. The Y-axis represents the log_10_ of the P-Value by SNP.

Figure S3 GWAS results of progressive sperm motility (MS) in Holstein bulls of China. Each dot represents one SNP. The X-axis represents 29 autosomes, respectively. The Y-axis represents the log10 of the P-Value by SNP.

Figure S4 GWAS results of number of motile sperm (NMSP) in Holstein bulls of China. Each dot represents one SNP. The X-axis represents 29 autosomes, respectively. The Y-axis represents the log10 of the P-Value by SNP.

Figure S5 GWAS results of number of sperms per ejaculate (NSP) in Holstein bulls of China. Each dot represents one SNP. The X-axis represents 29 autosomes, respectively. The Y-axis represents the log10 of the P-Value by SNP.
