# Supplementary figures and images for: Weighted Single-Step Genome-Wide Association Study of Semen Traits in Holstein Bulls of China
Source: Front Genet. 2019 Oct 25;10:1053. doi: 10.3389/fgene.2019.01053 (PMC6842931; doi:10.3389/fgene.2019.01053)

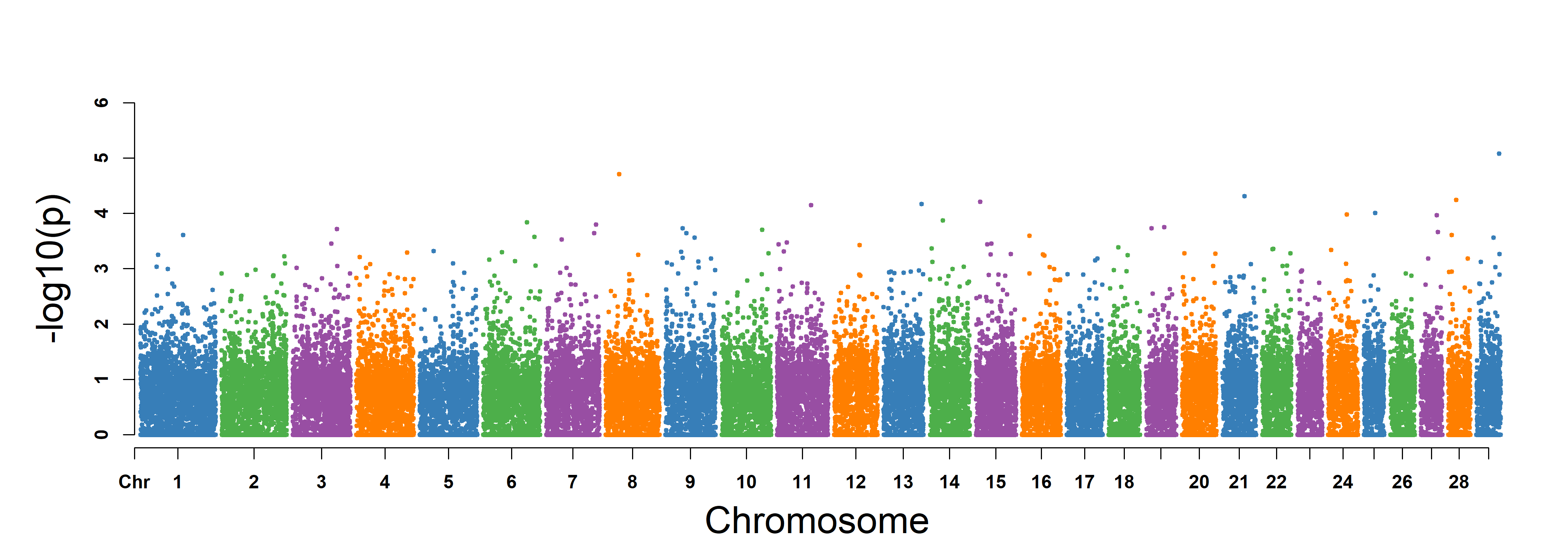

Supplement: Figure S1 — GWAS results of ejaculate volume (VE) in Holstein bulls of China. Each dot represents one SNP. The X-axis represents 29 autosomes, respectively. The Y-axis represents the log10 of the P-Value by SNP. [file Image_1.tiff]

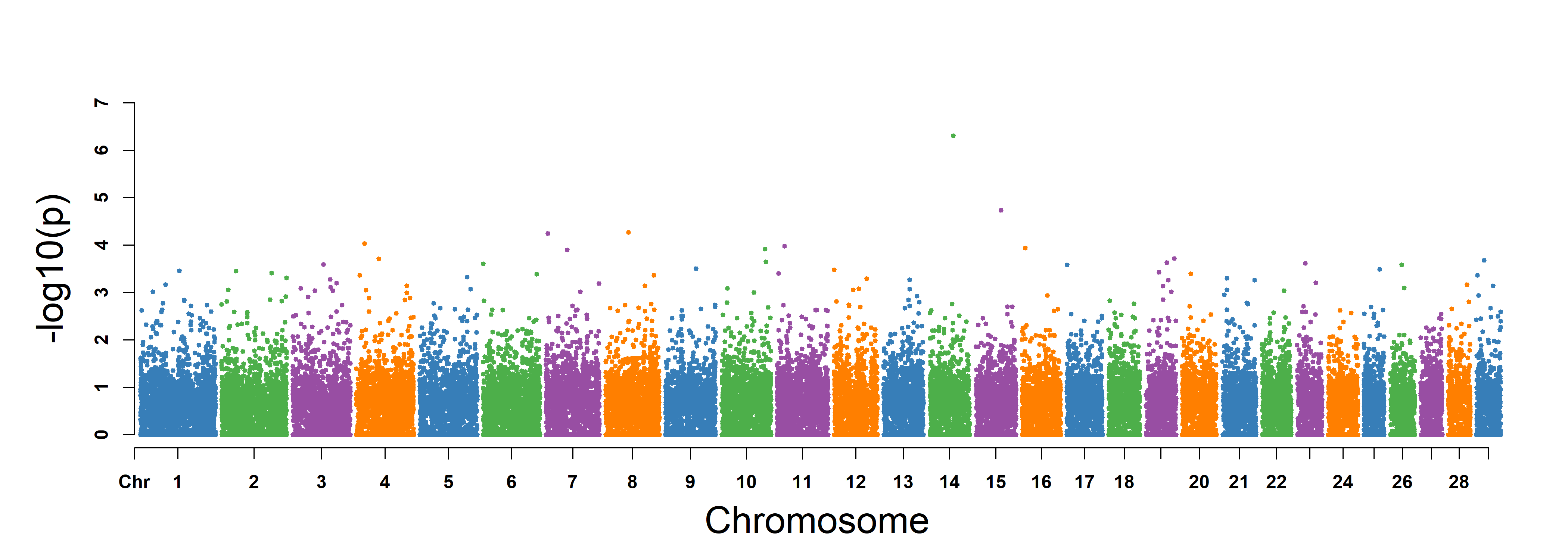

Supplement: Figure S2 — GWAS results of sperm concentration (SC) in Holstein bulls of China. Each dot represents one SNP. The X-axis represents 29 autosomes, respectively. The Y-axis represents the log10 of the P-Value by SNP. [file Image_2.tiff]

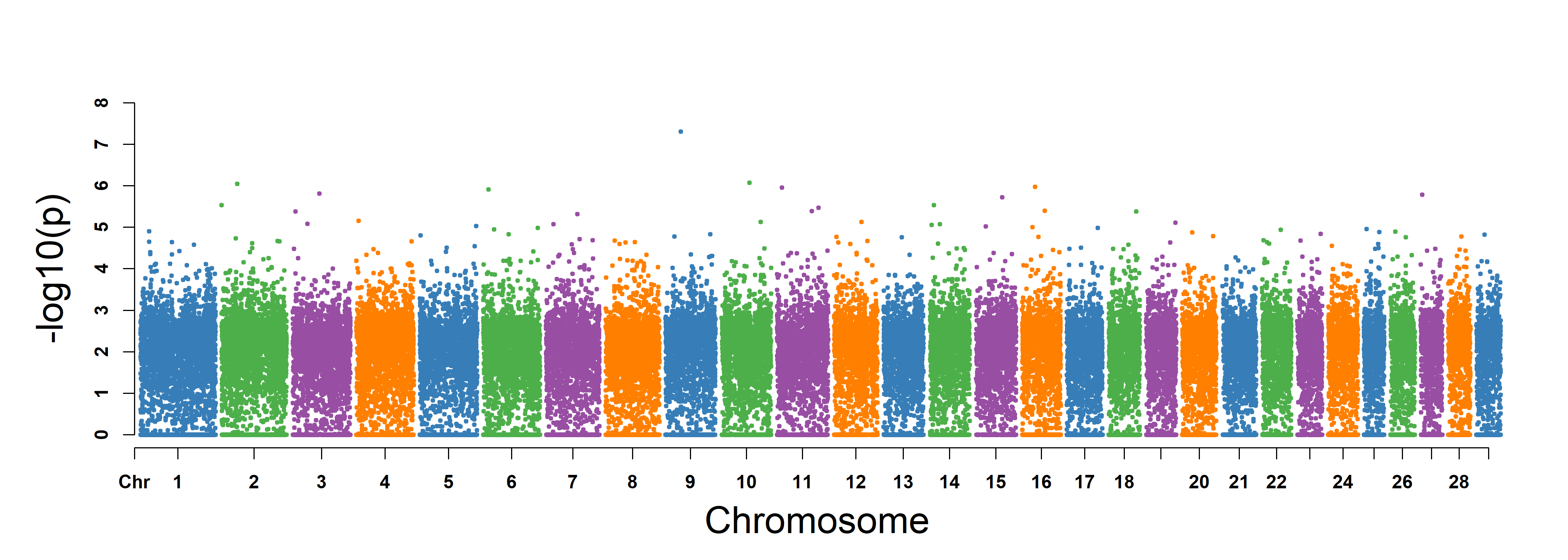

Supplement: Figure S3 — GWAS results of progressive sperm motility (MS) in Holstein bulls of China. Each dot represents one SNP. The X-axis represents 29 autosomes, respectively. The Y-axis represents the log10 of the P-Value by SNP. [file Image_3.tiff]

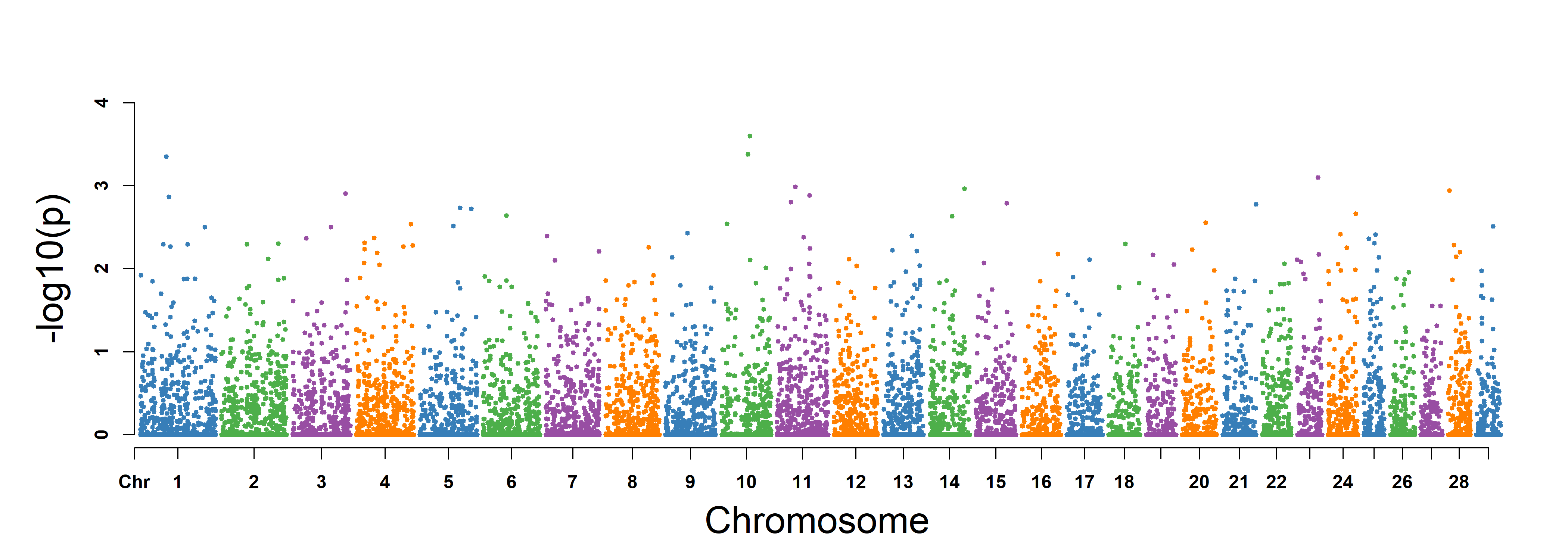

Supplement: Figure S4 — GWAS results of number of motile sperm (NMSP) in Holstein bulls of China. Each dot represents one SNP. The X-axis represents 29 autosomes, respectively. The Y-axis represents the log10 of the P-Value by SNP. [file Image_4.tiff]

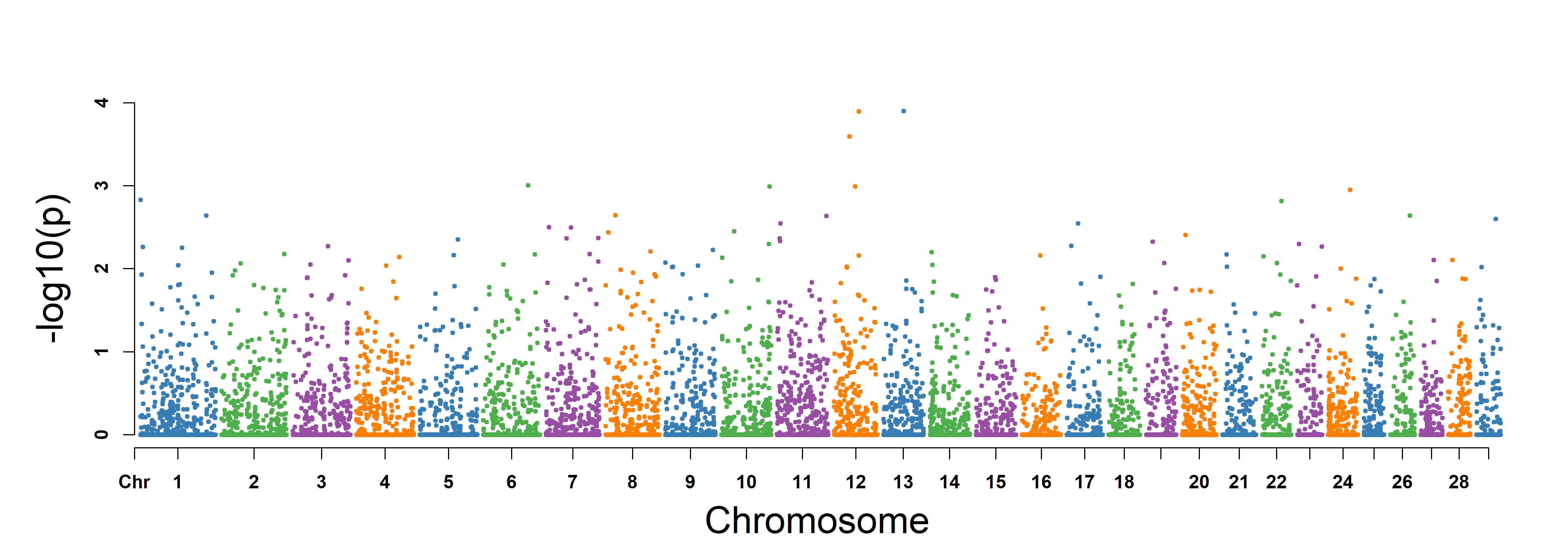

Supplement: Figure S5 — GWAS results of number of sperms per ejaculate (NSP) in Holstein bulls of China. Each dot represents one SNP. The X-axis represents 29 autosomes, respectively. The Y-axis represents the log10 of the P-Value by SNP. [file Image_5.tiff]
